# Complications, readmission and reoperation rates in one-stage bilateral versus unilateral total hip arthroplasty: a high-volume single center case–control study

**DOI:** 10.1038/s41598-021-85839-6

**Published:** 2021-03-18

**Authors:** Mattia Loppini, Alessandro Pisano, Cecilia Eugenia Gandolfi, Emanuela Morenghi, Guido Grappiolo

**Affiliations:** 1 Department of Biomedical Sciences, Humanitas University, Via Rita Levi Montalcini 4, Pieve Emanuele, 20090 Milan, Italy; 2 IRCCS Humanitas Research Hospital, Via Manzoni 56, Rozzano, 20089 Milan, Italy; 3 Fondazione Livio Sciutto Onlus, Campus Savona, Università Degli Studi di Genova, Via Magliotto 2, 17100 Savona, Italy

**Keywords:** Risk factors, Outcomes research

## Abstract

The study aimed to assess the safety of one-stage bilateral total hip arthroplasty (THA) compared with unilateral THA. In this retrospective observational case–control study were included patients undergoing unilateral (group 1) and one-stage bilateral (group 2) THA in a high-volume center. The groups were matched for gender, age at surgery, and pre-operative American Society of Anesthesiology score. The following variables were assessed: local and systemic complications, postoperative anemia, 30-day and 1-year readmission and reoperation rates, length of hospital stay, and ambulation time. Group 1 reported a significantly higher rate of local and systemic complications compared with group 2 (5.4% versus 3.9% and 29.6% versus 4.7%, respectively). Postoperative anemia was significantly lower in group 1 compared with group 2 (8.1% versus 30%). There was no significant difference in terms of 30-day and 1-year readmission rates between the two groups. The average length of hospital stay was 5.1 ± 2.3 days in group 1, and 5.3 ± 1.9 days in group 2 (p = 0.78). Ambulation time was significantly lower for group 1 (day 0.9 ± 0.9 in group 1, and day 1 ± 0.8 in group 2, p = 0.03). In a high-volume center, one-stage bilateral THA is a safe procedure compared with unilateral THA in terms of postoperative local and systemic complications, 30-day readmission and 1-year reoperation rates, and length of hospital stay.

## Introduction

Hip osteoarthritis (OA) is the most common orthopedic disorder worldwide with an estimated prevalence of 7.7% in the Italian population above 65 years of age, occurring bilaterally in 42% of cases^1^. In patients with bilateral hip OA, one-stage bilateral total hip arthroplasty (BTHA) has been shown to have several benefits over staged BTHA, including a reduction in hospitalization and rehabilitation times, the exposure to a single anesthetic procedure, fewer lost working days and lower costs^2^, and increased postoperative functional improvements^3–5^.

Although the frequency of one-stage BTHA is increasing in several countries^6^, the safety of this procedure remains controversial due to conflicting findings in the literature. In previous studies, one-stage BTHA demonstrated a higher risk of systemic and local complications^7,8^, autologous and allogenic blood transfusions^9,10^, increased length of stay^8^ and discharge to rehabilitation facilities^8–10^ compared to unilateral THA (UTHA). Among the same authors however, controversy existed. Some of them indeed, reported no significant differences in mortality and blood transfusions^8^, length of stay^9,10^, and 30-day major complications and readmissions or revision surgeries^10,11^. Moreover, the vast majority of the previous studies are based on national databases^7,8,10,11^. Despite they allow to include large study populations from numerous hospitals, they are affected by some bias such as the different perioperative management, application of the drug protocols, types of implants, and number of procedures per surgeon. Therefore, further independent cohort studies are required to investigate the safety of this procedure.

The present study aimed to assess the safety of one-stage BTHA compared with UTHA, in terms of local and systemic complications, 30-day and 1-year readmission and reoperation rates, length of hospital stay, and ambulation time in a single high-volume center.

## Methods

### Ethical approval

The present retrospective observational case–control study used medical records of patients included in a registry of orthopedic surgical procedures. The study protocol for the development of this registry was approved by the Ethical Committee of Humanitas Research Hospital (Approval number 618/17) and in strict accordance with the Helsinki Declaration, and good clinical practice guidelines. All individual participants signed a written informed consent before the surgical procedure and a written informed consent to be included in the registry of orthopedic surgical procedures.

In the present study were included patients undergoing UTHA (group 1) or one-stage BTHA (group 2) in our Institution from January 2015 to December 2016. These groups were matched for gender, age at surgery, and preoperative American Society of Anesthesiology (ASA) score. The exclusion criteria for bilateral one-stage BTHA were, patients with heart disease, left ventricular ejection fraction ≤ 40%, BMI > 40, hemoglobin (Hb) < 11 g/dL, severe CKD (GFR = 15-29 mL/min), and severe COPD (30% ≤ FEV_1_ ≤ 49%). The same exclusion criteria were applied to the matched control patients. Preoperative diagnosis included primary OA, secondary OA to mild hip dysplasia or epiphysiolysis or Perthes disease, and avascular necrosis of the femoral head.

The surgery was performed through a posterolateral approach with femur first technique^12^ with a combined spinal-epidural analgesia. In all patients, the tranexamic acid was administered in a dose of 1 g within one hour before surgery. Intraoperative cell saver suction was routinely used for all one-stage BTHA, and the blood collected during surgery was immediately reinfused afterwards. Uncemented implants were used in all surgeries. In both groups, Foley catheter and drains were placed and removed in day 1 after surgery. A standard antibiotic prophylaxis with cefazolin or clindamycin was used. Anticoagulation prophylaxis consisted of low-molecular-weight heparin (LMWH) beginning the night of surgery and continuing for 30 days postoperatively together with compression stockings.

Patients underwent the same rehabilitation protocol including walking with crutches and partial weight bearing, and strengthening exercises for abductor muscles. According to clinical practice, all patients underwent clinical and radiographic examination before and immediately after surgery. The follow-up visits with radiological examinations were performed at 1, 3, 6 and 12 months after surgery, then every two years.

The primary endpoints were the local and systemic complications occurring before the discharge to home or rehabilitation. Local complications are those occurring at the level of the surgical site, and included hematoma, superficial infection, deep infection, fracture, dislocation, and contact dermatitis associated with compression stockings. Systemic complications included deep vein thrombosis, pulmonary embolism and other pulmonary complications, gastrointestinal, neurological, and genitourinary complications. In addition, we also measured the occurrence of postoperative anemia. It was defined by a postoperative Hb ≤ 8 g/dL in third day after surgery or blood transfusions performed within the second postoperative day. The threshold values for transfusing were based on the Patient Blood Management (PBM) guidelines: Hb < 7 g/dL; Hb < 8 g/dL in cardiopathic patients; Hb < 8 g/dL in symptomatic patients (thoracic pain, orthostatic hypotension, tachycardia)^13^. The number of patients who underwent blood transfusions (both autologous and/or allogenic) and the number of blood units transfused intraoperatively up to the discharge were also recorded.

The secondary endpoints were 30-day and 1-year readmission and reoperation rates, length of hospital stay, and ambulation time. The readmission was defined as any unplanned readmission to any hospital within 30 days and 1 year after surgery. The length of hospital stay was measured as the number of days between the admission in the preoperative day and the day of hospital discharge to home or rehabilitation. In terms of ambulation recovery, patients were divided according their ability to walk the same day of surgery or the first postoperative day or the next postoperative days after surgery.

In addition, the results were adjusted for potential confounders including: BMI (categorized into two classes: not overweight, BMI ≤ 25 and overweight, BMI > 25), smoke (pack/years), alcohol status (units/day), preoperative anemia (Hb < 13 g/dL in males and < 12 g/dL in females), preoperative anticoagulant/antiaggregant therapy (yes or no), and Charlson Comorbidity Index (CCI) that predicts the one-year mortality for a patient who may have comorbid conditions^14^.

### Statistical analysis

Data are described as number and percentage, if categorical, or mean and standard deviation, if continuous. Differences between unilateral and one-stage bilateral THA group were explored with chi-square test if categorical, or with t-student test if continuous Gaussian distributed, or Mann Whitney otherwise.

The association between risks factors and local complications were explored with univariable logistic regression. All independent variables with a p value under 0.2 were then submitted to a backward multivariable logistic regression. The same analysis was performed for systemic complication, 30-day and 1-year readmission and reoperation rate, and postoperative anemia. A p value under 0.05 was considered as significative. All analyses were performed with stata15.

## Results

A total of 558 one-stage BTHAs performed in 279 patients (males/females = 171/108) (group 2) were matched with 521 UTHAs (males/females = 316/205) (group 1). The average age was 55 years both groups. The mean BMI was 27.2 ± 3.2 in group 1 and 26.5 ± 3.8 in group 2 (p = 0.08). The ASA score was I in 142 and 76 patients in group 1 and 2 respectively; II in 358 and 192 patients in group 1 and 2 respectively; III in 21 and 11 patients in group 1 and 2 respectively. The mean operative time was 61 ± 2.4 min in group 1 and 131 ± 2.9 min in group 2 (p < 0.001). In the BTHA group, the mean time between the two sides was 10 ± 3.2 min.

Systemic complications affected 28 (5.4%) patients in group 1 and 11 (3.9%) patients in group 2 (p = 0.46). Local complications affected 154 (29.6%) hips in group 1 and 26 (4.7%) hips in group 2 (p < 0.001). The number of local and systemic complications is reported in Table 1. Postoperative anemia was found in 42 patients (8.1%) of group 1 and 85 patients (30%) of group 2 (p < 0.001). Before the discharge to home or rehabilitation, the patients who received blood transfusions were 34 out of 521 (6.5%) in group 1, and 104 out of 279 (37.3%) in group 2 (p < 0.001). In group 1, the number of transfused blood units was one in 11 patients, and 2 in 23. In group 2, the number of transfused blood units was one in 86 patients, 2 in 14, and 3 in 4.

**Table 1:** Number of local and systemic complications in the two groups.

Local complications	Group 1 (521 hips)	Group 2 (558 hips)
Total	154	26
Hematoma	123 (80%)	20 (77%)
Superficial infection	2 (1%)	1 (4%)
Deep infection	1 (1%)	0
Fracture	1 (1%)	0
Dislocation	2 (1%)	0
Contact dermatitis	25 (16%)	5 (19%)

Systemic complications	Group 1 (521 patients)	Group 2 (279 patients)
Total	28	11
Deep vein thrombosis	0	0
Pulmonary embolism	0	0
Pulmonary complications	2 (7%)	1 (9%)
Gastrointestinal complications	9 (32%)	3 (27%)
Genitourinary complications	14 (50%)	6 (55%)
Neurological complications	3 (11%)	1 (9%)

In group 1, 3 patients returned in the operating room before the discharge: one for periprosthetic fracture, one for sciatic nerve compression due to hematoma, and one for acute preiprosthetic joint infection. In group 2, no patients returned in the operating room. The 30-day hospital readmissions were one for each group due to superficial wound infection managed with surgical debridement. At one year, only patient (0.2%) in group 1 was readmitted for stem loosening, managed with one-stage revision. In group 2, 5 patients (1.8%, p = 0.02) were readmitted at one year: two for stem loosening, managed with one-stage revision, two for periprosthetic calcification, managed with surgical removal, and one for angina. The reoperation rates were 0.7% and 0.3% at 30 days (p = 0.24), and 0.9% and 1.8% at 1 year (p = 0.3) in group 1 and 2 respectively.

The average length of hospital stay was 5.1 ± 2.3 days (range, 3 to 25) in group 1, and 5.3 ± 1.9 days (range, 4 to 14) in group 2 (p = 0.78). The number of patients discharged to rehabilitation was 477 and 246 in group 1 and 2 respectively (92.5% versus 88%, p = 0.13).

The average time of ambulation after surgery was 0.9 ± 0.9 and 1 ± 0.8 days in group 1 and 2 respectively (p = 0.03). In group 1, 200 (38.4%) patients ambulated on the same day of surgery, 207 (39.7%) on postoperative day 1, 93 (17.9%) on postoperative day 2, and the remaining 21 (4.0%) on postoperative day 3 or later. In group 2, 89 patients (31.9%) ambulated on the same day of surgery, 112 (40.1%) on postoperative day 1, 68 (24.4%) on postoperative day 2, and the remaining 10 (3.6%) on postoperative day 3 or later.

The association of risk factors with local complications are summarized in Table 2. After multivariable analysis local complications seem to be associated with bilateral procedure (OR = 0.29, 95%CI 0.19–0.45, p < 0.001), age (OR = 1.04, 95%CI 1.02–1.05, p < 0.001), and preoperative hemoglobin (OR = 1.36, 95%CI 1.18–1.57, p < 0.001).

**Table 2:** Univariable and multivariable logistic regression assessing risk factors for local complications.

	Univariable		Multivariable	
	OR (95% CI)	p value	OR (95% CI)	p value
Bilateral intervention	0.31 (0.20–0.47)	< 0.001	0.29 (0.19–0.45)	< 0.001
Gender (M)	1.75 (1.22–2.50)	0.002		
Age at surgery	1.03 (1.01–1.05)	< 0.001	1.04 (1.02–1.05)	< 0.001
**ASA**				
1	1			
2	1.07 (0.73–1.56)	0.726		
3	1.42 (0.62–3.28)	0.407		
Smoke	1.42 (1.01–2.01)	0.044		
Smoke pack-years*	1.01 (1.00–1.02)	0.042		
Alcohol units/day*	1.04 (0.97–1.13)	0.279		
**BMI**	1.04 (1.01–1.08)	0.013		
< 25	1			
25–30	1.28 (0.87–1.89)	0.209		
30–35	1.57 (0.96–2.57)	0.073		
> 35	1.96 (1.01–3.79)	0.046		
Dyslipidaemia	1.20 (0.77–1.87)	0.425		
Preoperative haemoglobin	1.27 (1.11–1.46)	< 0.001	1.36 (1.18–1.57)	< 0.001
Hypertension	1.34 (0.94–1.89)	0.105		
Hyperglycaemia	1.29 (0.67–2.50)	0.447		
Diabetes	1.18 (0.60–2.33)	0.624		
Thyroid comorbidities	0.84 (0.44–1.62)	0.614		
Osteoporosis	0.79 (0.22–2.79)	0.709		
Gastric comorbidities	1.12 (0.67–1.87)	0.662		
Hepatic comorbidities	1.22 (0.62–2.41)	0.564		
Chronic kidney disease	1.47 (0.38–5.76)	0.577		
Cardiological comorbidities	2.21 (1.23–3.95)	0.008		
Respiratory comorbidities	1.07 (0.61–1.86)	0.817		
Vascular comorbidities	1.66 (0.88–3.13)	0.120		
Pre-operative anaemia	0.48 (0.20–1.16)	0.103		
Blood comorbidities	0.42 (0.10–1.85)	0.252		
Rheumatic comorbidities	1.84 (0.87–3.90)	0.110		
HIV	0.82 (0.08–7.98)	0.867		
Charlson comorbidity index	1.22 (1.08–1.38)	0.001		

Due to the small of number of cases, no associations were explored for systemic complications, as well as 30-day and 1-year readmission and reoperation rates.

The association of risk factors with postoperative anemia is summarized in Table 3. After multivariable analysis anemia seem to be associated with bilateral procedure (OR = 7.39, 95%CI 4.63–11.80, p < 0.001), BMI (OR = 0.88, 95%CI 0.83–0.93, p < 0.001), preoperative hemoglobin (OR = 0.60, 95%CI 0.50–0.72, p < 0.001).

**Table 3:** Univariable and multivariable logistic regression assessing risk factors for postoperative anemia.

	Univariable		Multivariable	
	OR (95% CI)	p value	OR (95% CI)	p value
Bilateral intervention	4.91 (3.27–7.37)	< 0.001	7.39 (4.63–11.80)	< 0.001
Gender (M)	0.57 (0.39–0.83)	0.004		
Age at surgery	0.98 (0.96–0.99)	0.010		
**ASA**				
1	1			
2	1.05 (0.68–1.63)	0.815		
3	1.29 (0.49–3.39)	0.600		
Smoke (yes/no)	0.60 (0.39–0.94)	0.024		
Smoke pack-years*	0.98 (0.96–1.00)	0.037		
Alcohol units/day*	0.95 (0.86–1.05)	0.321		
**BMI**	0.85 (0.81–0.90)	< 0.001	0.88 (0.83–0.93)	< 0.001
< 25	1			
25–30	0.42 (0.27–0.64)	< 0.001		
30–35	0.18 (0.08–0.40)	< 0.001		
> 35	0.18 (0.06–0.61)	0.006		
Dyslipidaemia	0.98 (0.58–1.67)	0.954		
Preoperative haemoglobin	0.66 (0.57–0.77)	< 0.001	0.60 (0.50–0.72)	< 0.001
Hypertension	0.50 (0.31–0.80)	0.004		
Hyperglycaemia	0.75 (0.31 (1.81)	0.525		
Diabetes	0.93 (0.41–2.13)	0.868		
Thyroid comorbidities	1.08 (0.53–2.18)	0.839		
Osteoporosis	3.32 (1.18–9.31)	0.022		
Gastric comorbidities	0.98 (0.53–1.80)	0.957		
Hepatic comorbidities	0.96 (0.42–2.19)	0.919		
Chronic kidney disease	NC			
Cardiological comorbidities	4.52 (2.44–8.35)	< 0.001		
Respiratory comorbidities	0.54 (0.21–1.38)	0.198		
Vascular comorbidities	0.52 (0.23–1.15)	0.106		
Pre-operative anaemia	0.62 (0.24–1.60)	0.326		
Blood comorbidities	3.55 (1.34–9.33)	0.010		
Rheumatic comorbidities	0.35 (0.08–1.47)	0.150		
HIV	NC			
Charlson comorbidity index	0.83 (0.71–0.98)	0.028		

## Discussion

The main finding of the present study was that one-stage BTHA can be considered safe as UTHA in selected patients in terms of postoperative local and systemic complications, 30-day readmission and 1-year reoperation rates, and length of hospital stay.

The local complications resulted statistically significantly lower after one-stage BTHA compared with unilateral procedure (4.7% versus 29.6%). Moreover, the multivariate analysis demonstrated that bilateral procedure is a protective factor for local complications according with the OR value of 0.29, whereas age and preoperative haemoglobin can be considered mild risk factors due to an OR value slightly higher than 1. No significant difference was found in terms of systemic complications between the two groups. Previously, Rasouli et al.^7^ reported that one-stage BTHA is associated with a higher rate of systemic (3.52% versus 2.96%, P < 0.001) and local (4.96% versus 4.54%, P = 0.009) complications compared with UTHA. However, multivariate analysis demonstrated that BTHA was only associated with increased risk of systemic complications (OR: 2.1, P < 0.001). According to our findings, the greater rate of local complications in UTHA can be explained by taking into account hematoma that represented the 80% of the reported local complications. However, it is not clear why the incidence of the hematoma was higher in the UTHA compared with BTHA group. Because of the anticoagulation protocol was the same in the two groups, it may not have any effect in this respect. In a previous study, Tsay et al.^15^ also reported a double rate of hematoma in patients undergoing staged total knee arthroplasty compared with those undergoing simultaneous bilateral procedure. Finally, Glait et al.^8^ found for one-stage BTHA an increase in deep vein thrombosis (2.16% versus 0.84%, P < 0.0001) and pulmonary embolism (0.79 versus 0.37, P < 0.0001) compared to UTHA. On the other hand, both Morcos et al.^10^ and Stavrakis et al.^11^ reported that major complication rates were similar between the two groups.

In the present study the risk of blood transfusions was significantly higher after one-stage BTHA compared to UTHA (37.3% versus 6.5%). Previously, Parvizi et al.^9^ reported a higher allogenic transfusion rate in patients undergoing one-stage BTHA compared to those receiving UTHA (20% versus 10%, p = 0.001). However, all patients undergoing BTHA also received a predonated autologous blood unit during the surgical procedure. Morcos et al.^10^ reported a transfusion rate of 29.2% in the one-stage BTHA compared to 15.9% in the UTHA (p < 0.0001) taking into account any blood transfusion performed intraoperatively up to 72 h after surgery. In the present study, the higher rate of postoperative blood transfusions in BTHA can be explained by the inclusion of both autologous and allogenic transfusions performed during the hospital stay. As reported by previous studies^10,11^, no significant differences were found between the two groups in terms of 30-day readmissions or revision surgeries. The 1-year reoperation rates were also not significantly different between the two groups.

Previous studies reported not significant difference in terms of length of stay between one-stage BTHA and UTHA with an average of 4 days according to Parvizi et al.^9^ and 3 days according to Morcos et al.^10^ On the other hand, Glait et al.^8^ showed a significantly reduced length of stay for UTHA compared to one-stage BTHA (5.92 versus 9.06 days). However, all the studies demonstrated a higher rate of patients discharged to rehabilitation facility in the one-stage BTHA group (from 39 to 96%) compared with UTHA group (from 21 to 74%)^8–10^. In the present study, the bilateral procedure was not associated with a longer hospital stay according with the literature. The average value of length of stay (5 days in both groups) higher than previous studies^9,10,16,17^ can be explained by the admission the day before of surgery for all patients, whereas in the previous studies patients were admitted the day of surgery. Moreover, the long hospital stay (> 14 days) in some patients of the UTHA group can be explained by the postoperative complications resulting in reoperation before the discharge. Finally, the no significant difference in the rate of rehabilitation versus home discharge between the two groups can be explained by the higher number of patients with UTHA discharged to rehabilitation facility available in the hospital.

Strengths of this study include the large number of patients operated on by the same team of experienced, high volume orthopedic surgeons throughout a very restricted index period of time (24 months). This allowed the surgical technique, as well as anesthesiologic and postoperative care protocols to remain unaltered, and thus comparable between the two groups, throughout the whole time period. Moreover, standardized triggers for transfusion have been applied preventing bias in the indication for postoperative blood transfusions. Finally, since it is a single-center study based on clinical records of the hospital database, a wider number of potential confounders and peri-operative information were taken into account.

The present study has some limitations. First of all, it is a retrospective observational case–control study. Nevertheless, relevant potential confounders were taken into account by performing a logistic regression analysis in order to minimize the impact of bias. Second, the blood loss estimation did not take into account the volume of blood loss during surgery and the volume of blood collected into drains. Moreover, in all bilateral procedures the blood collected with the intraoperative cell saver suction was immediately reinfused after surgery. Therefore, the effect of this procedure on the postoperative anemia cannot be evaluated. Third, no standardized criteria have been applied for the indication to home versus rehabilitation discharge. Finally, the results of the present study could be not representative of other high-volume centers operating under different types of healthcare systems other than a government-managed facility.

In conclusion, in a high-volume center, one-stage bilateral THA is a safe procedure compared with unilateral THA in terms of postoperative local and systemic complications, 30-day readmission and 1-year reoperation rates, and length of hospital stay.
